# Comparison of Carrier and *de novo* Pathogenic Variants in a Chinese DMD/BMD Cohort

**DOI:** 10.3389/fneur.2021.714677

**Published:** 2021-08-05

**Authors:** Jinfu Lin, Huan Li, Ziyu Liao, Liang Wang, Cheng Zhang

**Affiliations:** ^1^Department of Neurology, The First Affiliated Hospital, Sun Yat-sen University, Guangzhou, China; ^2^Guangdong Provincial Key Laboratory of Diagnosis and Treatment of Major Neurological Diseases, National Key Clinical Department and Key Discipline of Neurology, Guangzhou, China

**Keywords:** Duchenne muscular dystrophy, Becker muscular dystrophy, *DMD* gene, carrier variants, *de novo* variants, carrier frequency

## Abstract

**Background:** Duchenne and Becker muscular dystrophy (DMD/BMD) are X-linked recessively inherited neuromuscular disorders caused by deletions, duplications, or small mutations in the *DMD* gene. With advances in prenatal diagnosis decreasing the number of affected offspring from carrier mothers, the frequency of *de novo* variants could increase. Therefore, determining the differences between the carrier and *de novo* variants of the *DMD* gene, which are rarely explored, is important for trial planning and genetic diagnosis in the future.

**Methods:** A total of 440 patients, 349 of whom had DMD and 91 had BMD, diagnosed in our department between 2012 and 2019, along with their respective mothers, were included in this study. Multiplex ligation-dependent probe amplification was used to detected deletions and duplications in patients and their mothers. Small mutations were detected using next-generation sequencing in the patients, followed by Sanger sequencing in the mothers.

**Results:** Deletions, duplications, and small mutations were identified in 204, 46, and 99 of the 349 patients with DMD and in 50, 10, and 31 of the 91 patients with BMD, respectively. *De novo* deletions were more concentrated in hotspot regions than carrier deletions of DMD/BMD. No clear bias was observed in the variant distribution between carriers, *de novo* duplications, and small mutations in DMD/BMD. The carrier frequency of DMD (61.6%) was lower than that of BMD (69.2%), but the difference was not statistically significant. The carrier frequency of deletions of the *DMD* gene (51.2%) was significantly lower than those of duplications (75%) and small mutations (81.5%).

**Conclusion:** Compared to *de novo* deletions, deletions from carrier mothers had a wider distribution. Moreover, there was no significant difference between the carrier frequencies of DMD and BMD. Duplications and small mutations were more commonly inherited, while deletions were present *de novo*.

## Introduction

Duchenne muscular dystrophy (DMD) is an X-linked recessively inherited fatal muscle disease, with an incidence of 15.9–21.9 for every 100,000 live newborn males (1–3). It is characterized by progressive weakness and muscle atrophy, accompanied by pseudohypertrophy of the gastrocnemius and a positive Gowers sign. Patients usually die of cardiorespiratory failure in the second or third decade of life. Pathogenic variants, such as deletions, duplications, and small mutations, in the *DMD* gene encoding dystrophin account for both DMD and Becker muscular dystrophy (BMD), a milder form of the disease with later onset and slower progression.

While DMD and BMD are usually diagnosed in men and rarely in women, many female carriers are asymptomatic but have a 50% risk of giving birth to male offspring with the disease. Previous studies have noted a different distribution of mutation types between *de novo* and carrier pathogenic variants (4, 5). With recent advances in molecular diagnostics that allow prenatal diagnosis, helping to identify female carriers before their first births, carrier mothers can be prevented from giving birth to offspring with DMD/BMD, consequently affecting the distribution of DMD/BMD pathogenic variants. Because of this, specific gene therapies, such as exon skipping therapy, could also be affected and might need to be changed. Therefore, more evidence on the difference between carrier and *de novo* pathogenic variants is needed. Hence, this study aimed to analyze the *DMD* gene variants in 440 patients with DMD/BMD, along with their respective mothers, and explore the difference.

## Materials and Methods

### Study Design and Participants

From January 2012 to June 2019, 440 Chinese male patients from independent families were diagnosed with DMD or BMD in our department based on clinical characteristics, serum creatine phosphokinase detection, and molecular genetic analysis. The diagnosis was based on disease severity, such as the age at which ambulation was lost (DMD <12 years old, BMD ≥12 years old). For patients with ambulation, those with obvious muscle weakness before 5 years of age were classified as having DMD, while those with a later onset, very mild motor dysfunction, and considerably longer survival were classified as having BMD. All clinical diagnoses were confirmed using molecular genetic analysis. The protocol of this study was approved by The Ethics Committee of the First Affiliated Hospital, Sun Yat-sen University.

### Molecular Genetic Analysis

All patients and their mothers provided informed consent before molecular genetic analysis. Peripheral blood was drawn from the patients and their mothers, and DNA was extracted using a standard procedure. Multiplex ligation-dependent probe amplification (MLPA) was used to detect deletions and duplications in patients and their mothers (6). Patients with negative MLPA results were examined for small mutations using next-generation sequencing as previously described, and Sanger sequencing was then used to detect whether the mothers carried the small mutation of the *DMD* gene (6, 7).

### Statistical Analysis

In the analysis of deletions, the starting and termination exons of a deletion were considered as the deleted ends. For example, for exon 45–52 deletion, the deleted ends were exons 45 and 52. The Chi-square test and Fisher's exact test were used for statistical analyses, and differences were considered statistically significant at *P* < 0.05.

## Results

### Distribution of Pathogenic Variants Among Patients With DMD and BMD

**Figure 1:**
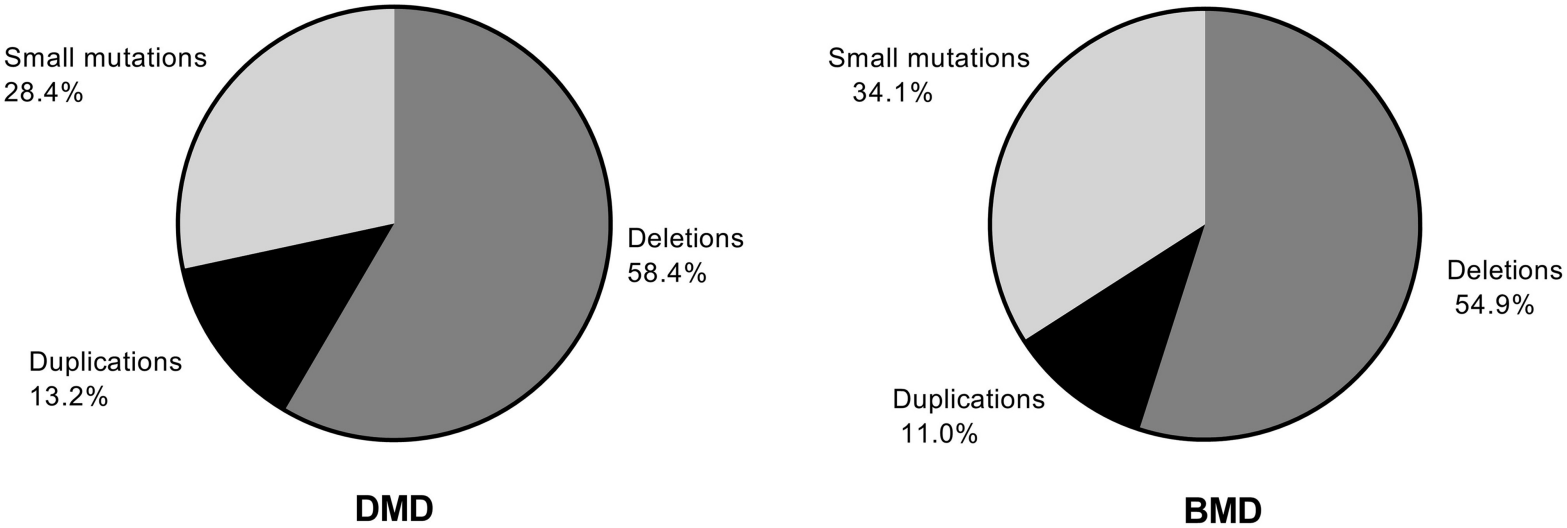
Distribution of deletions, duplications, and small mutations in patients with Duchenne muscular dystrophy (DMD) and Becker muscular dystrophy (BMD).

Among 349 patients with DMD and 91 with BMD, deletion was the most common variant type, with 204 and 50 deletions for DMD and BMD, respectively, followed by small mutations, with 99 and 31 mutations, respectively. Duplication was the least frequent type, with 46 and 10 duplications for DMD and BMD, respectively. The constitution of the DMD variants is similar to that of the BMD variants (Figure 1).

### Difference Between the Carrier and *de novo* Variants in Patients With DMD

**Figure 2:**
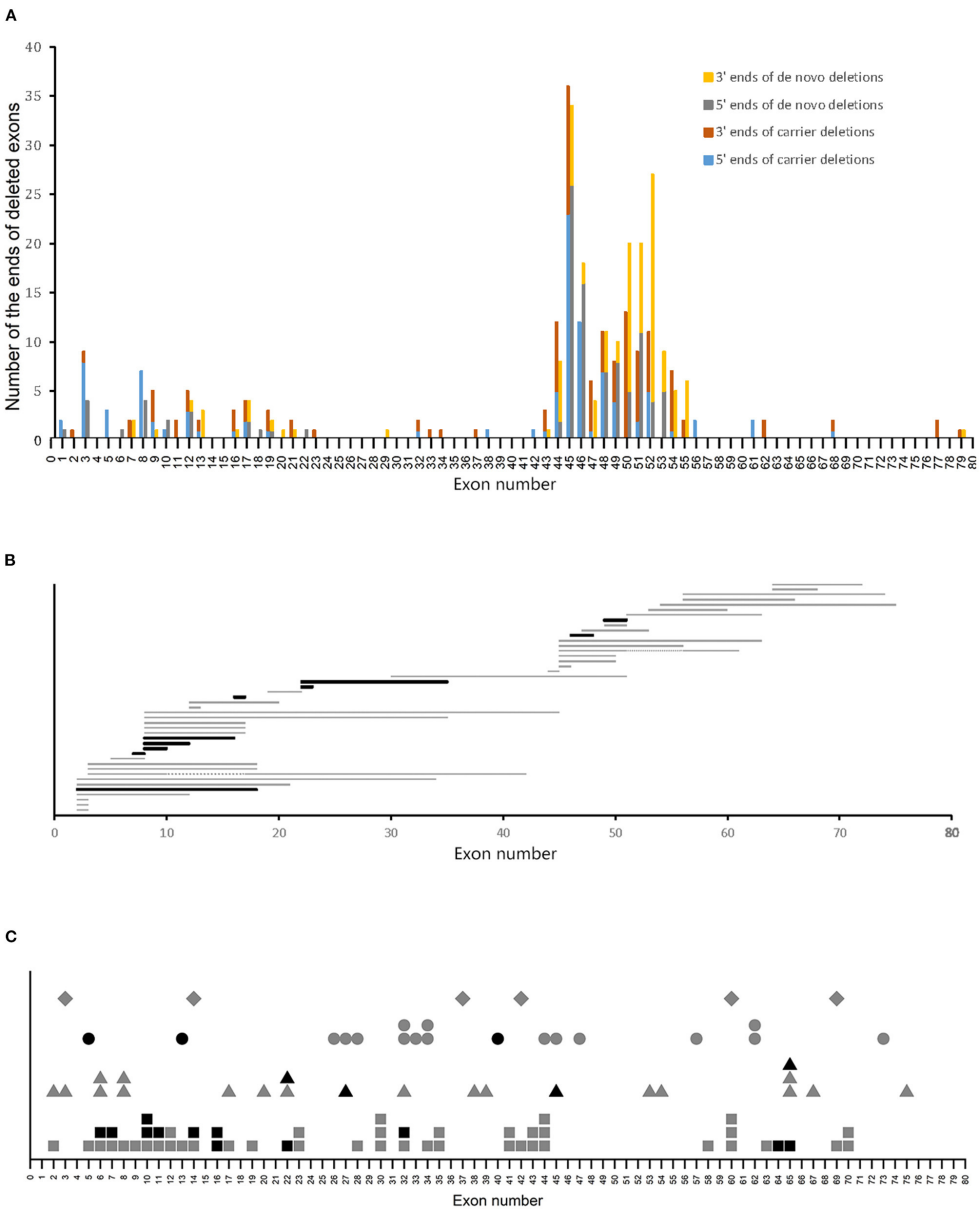
Variant sites in patients with DMD. **(A)** Distribution of ends of deletions in patients with DMD. **(B)** Distribution of duplications in patients with BMD. Horizontal bars represent the duplicated regions. Gray and black bars represent carrier and *de novo* variants, respectively. Two patients with carrier duplications had two duplicated regions each, and the two duplicated regions were connected with gray dotted lines. **(C)** Distribution of small mutations. Gray and black symbols represent carrier and *de novo* variants, respectively. Squares, triangles, circles, and rhombus represent non-sense mutations, splice site mutations, small deletions/insertions, and missense mutations, respectively.

We investigated the relationship between variant sites and the carrier status of the mothers of patients with DMD. Of the 204 deletions in the *DMD* gene observed in patients with DMD, 100 were carrier variants, and 104 were *de novo* variants. Deleted ends were mainly located at exons 3, 8, 12, 17, and 44–55, which account for 76 and 90.4% of the total deleted ends, respectively. However, the deleted ends of patients with carrier variants had a wider distribution than those with *de novo* variants (Figure 2A).

A previous study revealed that most of the deletions in the *DMD* gene are located in exons 2–20 and 45–55, which were referred to as the proximal hotspot region and the distal hotspot region, respectively (8). In this study, 73% of the deletions in patients with carrier variants were located at the hotspot regions, and this was significantly lower than that in patients with *de novo* variants, of which 90.38% were in the hotspot regions (*P* < 0.05, using Chi-square test) (Table 1). Further analysis of the deletions in the hotspot regions revealed that compared to *de novo* deletions, carrier deletions tended to be more concentrated within the distal hotspot region. However, this difference was not statistically significant (*P* > 0.05, using the Chi-square test).

**Table 1:** Number of patients with DMD who had deletions located in the proximal, distal hotspot region and non-hotspot region.

	**Carrier deletions**	***de novo* deletions**	**Overall**
Proximal hotspot region	17	12	29
Distal hotspot region	56	82	138
Non-hotspot region	27	10	37
Overall	100	104	204

Regarding duplications, no clear difference in distribution was found between patients with carrier and *de novo* variants (Figure 2B). Notably, we found 99 sites of small mutations scattered across the *DMD* gene. However, no clear discrepancy in the distribution of small mutation sites was observed with respect to the carrier status of the mothers (Figure 2C).

### Difference Between the Carrier and *de novo* Variants in Patients With BMD

**Figure 3:**
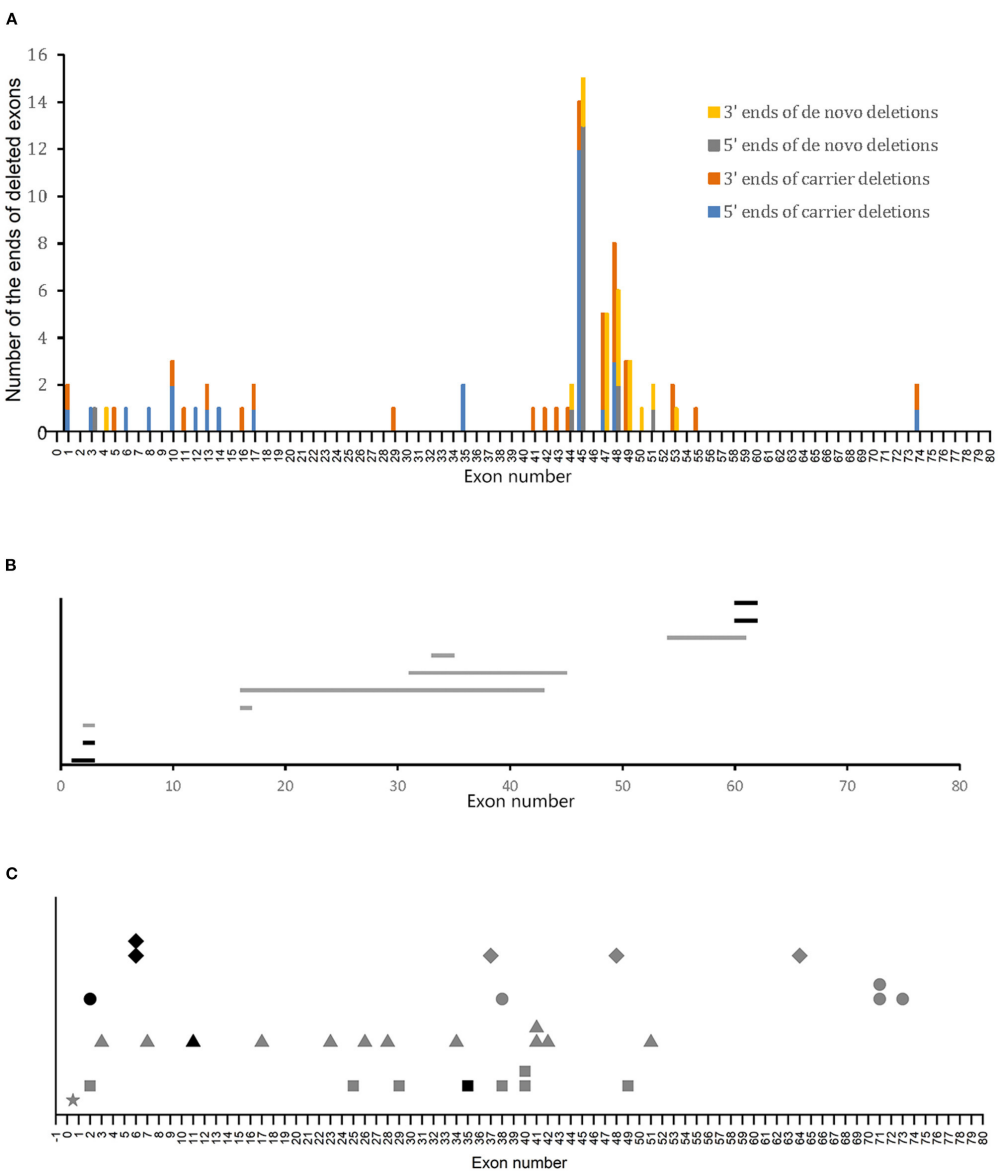
Variant sites in patients with BMD. **(A)** Distribution of deletion ends in patients with BMD. **(B)** Distribution of duplications in patients with BMD. Horizontal bars represent the duplicated regions. Gray and black bars represent carrier and *de novo* variants, respectively. **(C)** Distribution of small mutations. Gray and black symbols represent carrier and *de novo* variants, respectively. Squares, triangles, circles, and rhombus represent non-sense mutations, splice site mutations, small deletions/insertions, and missense mutations, respectively. The star represents a point mutation (c.-54T>A) in the 5′-untranslated region (UTR).

Of the 50 deletions in the *DMD* gene observed in patients with BMD, 31 were carrier variants, while 19 were *de novo* variants. The most frequent ends of deletions were exons 45, 47, 48, and 49, accounting for 48.4 and 76.3% of the total deleted ends in the carrier and *de novo* variants, respectively. Carrier deletions had a wider distribution than *de novo* deletions (Figure 3A). Moreover, 74.2% (23/31) of carrier deletions and 100% (19/19) of *de novo* deletions were in the hotspot regions (Table 2), and this difference was statistically significant (*P* < 0.05, using Chi-square test). Further analysis of these deletions in the hotspot regions revealed that compared to carrier deletions (78.3%), *de novo* deletions tended to be more concentrated in the distal hotspot region (94.7%). However, this difference was not statistically significant (*P* > 0.05, using Fisher's exact test).

**Table 2:** Number of patients with BMD who had deletions located in the proximal, distal hotspot region and non-hotspot region.

	**Carrier deletions**	***de novo* deletions**	**Overall**
Proximal hotspot region	5	1	6
Distal hotspot region	18	18	36
Non-hotspot region	8	0	8
Overall	31	19	50

We next investigated the sites of duplications and small mutations and their relationship with the carrier status of BMD. Six of the 10 duplications and 26 of the 31 small mutations were carrier variants, which included a point mutation (c.-54T>A) in the 5′-untranslated region. No clear bias of variant sites of either duplications or small mutations was indicated between the carrier and *de novo* variants (Figures 3B,C).

### Difference in Carrier Frequencies Between Mothers of Patients With DMD and BMD

**Figure 4:**
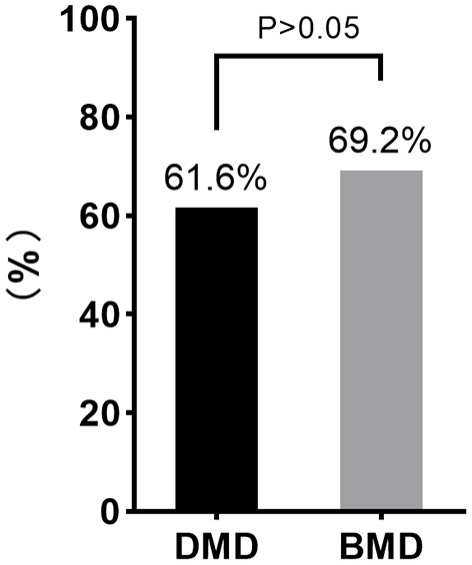
Comparison of the carrier frequency of DMD and BMD. No significant difference between the carrier frequencies of DMD and BMD was observed using the Chi-square test (*P* > 0.05).

Regarding carrier frequencies, a higher frequency of *DMD* mutation was observed in the mothers of patients with BMD, as only 215 of the 349 (61.6%) mothers of patients with DMD were causative mutation carriers, while 63 (69.2%) of the 91 mothers of patients with BMD carried the mutations. However, this difference between the carrier frequencies of DMD and BMD was not statistically significant (Figure 4).

### Carrier Frequencies in the Mothers of Patients With DMD/BMD According to Variant Types

**Figure 5:**
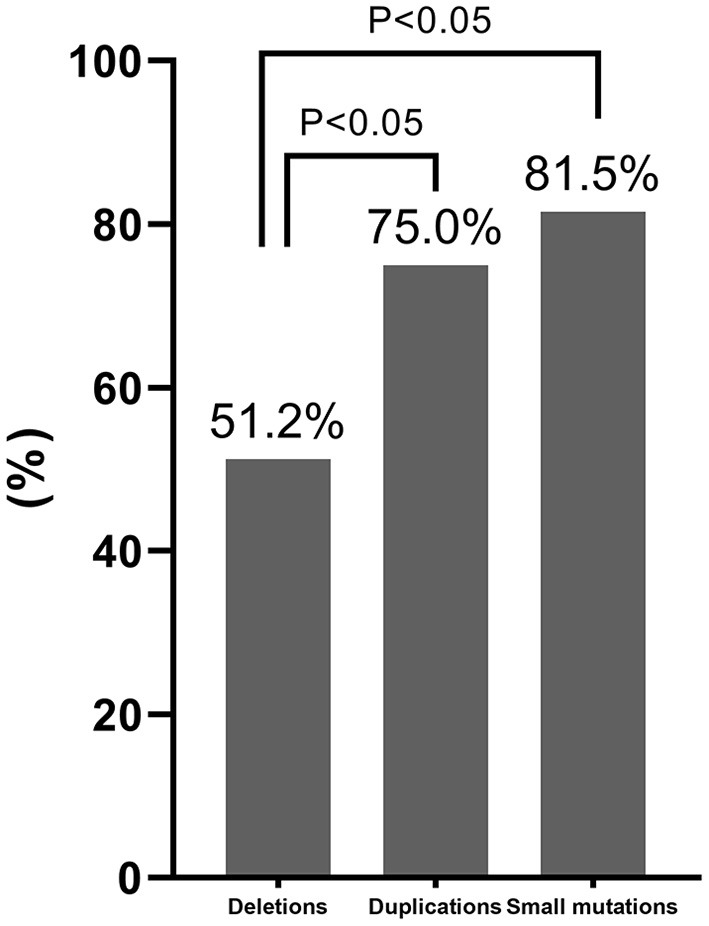
Comparison of the carrier frequency of different variant types. The carrier frequency of deletions was significantly lower than those of duplications and small mutations based on Chi-square test results (*P* < 0.05).

The carrier frequencies of deletions, duplications, and small mutations in mothers of patients with DMD/BMD were 51.2% (130/254), 75% (42/56), and 81.5% (106/130), respectively. The carrier frequency of deletions was significantly lower than those of duplications and small mutations (Figure 5).

## Discussion

In the present study, deletions were the most frequent mutations detected in both DMD and BMD, with small mutations being the second and duplications being the least frequent. The proportions of the three types of mutations in DMD and BMD were in accordance with previous reports (8, 9). In addition, the frequency of *de novo* mutations was 38.4 and 30.8% for DMD and BMD, respectively, with a 36.8% total frequency, which is consistent with previously reported frequencies ranging from 24 to 39.5% (6, 8, 10).

The present study revealed that compared to carrier variants, *de novo* variants were more concentrated in hotspot regions in both patients with DMD and BMD who had deletions. Furthermore, deletions from carrier mothers were scattered across the *DMD* gene. Previous reports by Ma et al. (10) and Lee et al. (11) revealed no significant difference in the distribution of deletion mutations from carrier and non-carrier mothers, perhaps owing to conclusions drawn from diagrams rather than quantitative and statistical analyses. Our study also indicated no significant difference in carrier frequency between deletion mutations in the proximal (58.6%, 17/29) and distal hotspot regions (40.6%, 56/138) in patients with DMD (Table 2), which is consistent with the report of Lee et al. (11). However, a trend of the carrier frequency of deletion mutations in the proximal being higher than that in distal hotspot regions was observed. Further research with a larger database is needed.

The theoretical frequency of DMD patients inheriting the mutations from carrier mothers is 2/3, as most patients with DMD do not survive long enough to produce offspring (12). In our study, the carrier frequency of DMD was 61.6%, which is slightly lower than the theoretical frequency. This may have resulted from including patients from independent families in the present study and the overall decrease in numbers of patients from carrier mothers due to advances in prenatal diagnosis. Meanwhile, the carrier frequency of BMD was 69.2% in the present study. As patients with BMD have milder symptoms, they can usually raise offspring; hence, mutations are more likely to pass on from a male patient to his daughters and on to his grandsons. Therefore, theoretically, the carrier frequency of BMD is higher than that of DMD. Nonetheless, in our study, the difference in carrier frequency between DMD and BMD was not statistically significant. This result is consistent with the report of Zimowski et al. (5) in a Polish population, in which the carrier frequency was 61.9% for DMD and 68.1% for BMD. A report by Toksoy et al. (13) in a Turkish population also revealed no statistically significant difference between the carrier frequency of DMD and BMD, with 45.5% for DMD and 42.9% for BMD. In contrast, Lee et al. (11) reported a significant difference in the carrier frequencies of DMD (57.6%) and BMD (89.5%) in a Japanese population, probably due to the small number of tested mothers (Table 3). In addition, the non-significant difference observed in our study may have been due to the recent advances in genetic diagnosis. BMD can be diagnosed more easily than ever before. While the clinical phenotype of BMD is highly variable and the onset of its symptoms varies from childhood to adulthood (14, 15), nearly 90% of patients with BMD will show the first symptoms by the age of 20 and are diagnosed by the age of 35 (16). Therefore, most patients with BMD will have been diagnosed before their daughters reach childbearing age. Moreover, with the help of prenatal diagnosis, the pathogenic variants will likely not be passed on to the grandsons.

**Table 3:** Comparison of carrier frequencies of DMD and BMD in different countries.

	**Tested mothers**	**Carrier frequency of DMD**	**Carrier frequency of BMD**	**Significant difference**
Poland (5)	744	377/609 (61.9%)	92/135 (68.1%)	No
Japan (11)	154	80/139 (57.6%)	17/19 (89.5%)	Yes
Turkey (13)	122	46/101 (45.5%)	9/21 (42.9%)	No
Our data	440	215/349 (61.6%)	63/91 (69.2%)	No

In the present study, the carrier frequency of deletions was significantly lower than those of duplications and small mutations in mothers of patients with DMD/BMD, consistent with the results of previous studies in Polish, Chinese, and Japanese populations, which also revealed a lower carrier frequency of deletions than those of duplications and small mutations (5, 10, 11, 17, 18). However, the difference in the reports by Lee et al. and Zhang et al. (11, 18) showed no statistical significance, probably due to the relatively small sample sizes. Recent studies from India and Turkey have also revealed that the carrier frequency of small mutations is significantly higher than that of deletions (4, 13) (Table 4). Duplications and small mutations in the *DMD* gene are more common during spermatogenesis, while deletions appear more commonly during oogenesis (19–21); hence, duplications and small mutations from spermatogenesis are inherited by daughters and then passed on to the grandsons and appear to be carrier mutations. However, deletions from oogenesis are either directly passed on to sons or are indirectly passed on to grandsons through carrier daughters and appear to be *de novo* or carrier mutations. This may account for the difference in the carrier frequencies of the three variant types.

**Table 4:** Carrier frequency of DMD/BMD based on different variant types in different countries.

	**Tested mothers**	**Carrier frequency of deletions**	**Carrier frequency of duplications**	**Carrier frequency of small mutations**	**From independent families**	**Clinical type of probands**
Poland (5)^*^	744	58.2%	77.9%	78.8%	Yes	DMD/BMD
Japan (11)	154	53.5%	66.7%	67.9%	Yes	DMD
India (4)^*^	91	47.8%	100%	64%	No	DMD
China (17)^*^	474	50.5%	81.3%	80.8%	Yes	DMD/BMD
China (18)	52	74.1%	100%	95%	Yes	DMD
China (10)^*^	442	59.8%	85.7%	78.9%	Yes	DMD/BMD
Turkey (13)^*^	138	31.0%	47.4%	68.6%	Yes	DMD, BMD, MF, and hyper-CKemia
Our data^*^	440	51.4%	75.0%	80.9%	Yes	DMD/BMD

* *Represents significantly lower carrier frequency of deletion. MF, Manifesting female*.

Notably, deletion ends were distributed more widely in the DMD gene carrier mutations than in *de novo* mutations. Additionally, 79.1% (170/215) and 71.4% (45/63) of carrier mothers had no significant family history in the DMD and BMD carrier groups, respectively. We discovered a difference in DMD gene mutations between the carriers with family history and the carriers without family history but found it statistically insignificant (Supplementary Figures 1, 2; Supplementary Table 1). As *de novo* mutations are derived from the oogenesis of mothers and carrier mutations are derived from spermatogenesis or oogenesis of maternal ancestors, there may be some differences between the distribution of deletions derived from spermatogenesis and oogenesis. However, further research is needed to explore the differences and their origins.

Distinguishing carrier status and prenatal diagnosis are of great importance to reduce DMD/BMD patient numbers. Mothers in our study were unaware of their carrier status until they and their affected children came to our department and went through genetic detection. The cases of three mothers with negative DMD mutation detection results of peripheral blood DNA were suspected as mosaicism because they gave birth to or were pregnant with more than one affected child or fetus with the same DMD mutation. However, the suspected mosaicism was not confirmed at the molecular level. There were 40 families in our study with multiple affected siblings in which the carrier mothers were unaware of their carrier status and gave birth to another one or more affected children without a prenatal diagnosis. Of the 31 follow-up prenatal diagnoses in our study, eight affected male fetuses were distinguished and aborted. A previous study in the Netherlands revealed through prenatal diagnosis, the percentage of the first affected boys increased from 62% in 1961–1974 to 88% in 1993–2002 (22). To prevent carrier mothers from giving birth to more affected children, it is essential to make an early diagnosis of DMD/BMD probands, for instance, by introducing DMD newborn screening (1, 23). Most of the DMD and BMD carrier mothers did not have a family history in our study, and it was difficult to establish their carrier status before their first birth. Therefore, it is still difficult to prevent carrier mothers without a family history from giving birth to a first affected child unless newborn screening of DMD/BMD carriers is introduced in the future.

The limitations of our study were that it was a single-center study revealing only the experience of our hospital. Moreover, the sample size is relatively small, and further researches with larger databases are needed.

In conclusion, our study revealed a lower carrier frequency of deletions compared to duplications and small mutations of the DMD gene. Moreover, compared to carrier variants, *de novo* variants were more concentrated within the hotspot regions in DMD/BMD patients who had deletions. The results highlight the variability of the mutation spectrum of the *DMD* gene attributable to a decreasing number of affected offspring from carrier mothers. Up to date research focusing on pathogenic variants spectrum of DMD/BMD patients is of vital importance for research in specific genetic therapy.

## Data Availability Statement

The raw data supporting the conclusions of this article will be made available by the authors, without undue reservation.

## Ethics Statement

The studies involving human participants were reviewed and approved by The Ethics Committee of the First Affiliated Hospital, Sun Yat-sen University. Written informed consent to participate in this study was provided by the participants' legal guardian/next of kin.

## Author Contributions

JL designed the study, analyzed the data, and drafted the manuscript. HL, ZL, and LW assisted in data acquisition. CZ assisted in data analysis and in revising the manuscript critically for important intellectual content. All authors contributed to the article and approved the submitted version.

## Conflict of Interest

The authors declare that the research was conducted in the absence of any commercial or financial relationships that could be construed as a potential conflict of interest.

## Publisher's Note

All claims expressed in this article are solely those of the authors and do not necessarily represent those of their affiliated organizations, or those of the publisher, the editors and the reviewers. Any product that may be evaluated in this article, or claim that may be made by its manufacturer, is not guaranteed or endorsed by the publisher.

## Abbreviations

BMD: Becker muscular dystrophy
DMD: Duchenne muscular dystrophy
MLPA: Multiplex ligation-dependent probe amplification.
