# Supplementary material for: Comparison of Carrier and de novo Pathogenic Variants in a Chinese DMD/BMD Cohort
Source: Front Neurol. 2021 Aug 5;12:714677. doi: 10.3389/fneur.2021.714677 (PMC8375267; doi:10.3389/fneur.2021.714677)
Supplement: Supplementary file 1 [file Table_1.DOCX]

Supplementary table 1. Number of DMD carrier mothers who had deletions located in the proximal, distal hotspot region and non-hotspot region（analyzed using Chi-square test）

|  | Carrier mothers with family history | Carrier mothers without family history | Overall |
| --- | --- | --- | --- |
| Proximal hotspot region | 3 | 14 | 17 |
| Distal hotspot region | 12 | 43 | 55 |
| Non-hotspot region | 4 | 24 | 28 |
| Overall | 19 | 81 | 100 |

Supplementary table 2. Number of BMD carrier mothers who had deletions located in the proximal, distal hotspot region and non-hotspot region（analyzed using Fisher’s exact test）

|  | Carrier mothers with family history | Carrier mothers without family history | Overall |
| --- | --- | --- | --- |
| Proximal hotspot region | 1 | 4 | 5 |
| Distal hotspot region | 6 | 12 | 18 |
| Non-hotspot region | 2 | 6 | 8 |
| Overall | 9 | 22 | 31 |

Supplementary Figure 1. Variant sites in carrier mothers of DMD patients. **(A**) Distribution of ends of deletions in carrier mothers of DMD patients. **(B)** Distribution of duplications in carrier mothers of DMD patients . Horizontal bars represent the duplicated regions. Gray and black bars represent carrier mothers with and without family history, respectively. Two carrier mothers of DMD patients with duplications had two duplicated regions each, and the two duplicated regions were connected with dotted lines. **(C)** Distribution of small mutations. Gray and black symbols represent carrier mothers with and without family history, respectively. Squares, triangles, circles, and rhombus represent nonsense mutations, splice site mutations, small deletions/insertions, and missense mutations, respectively.

Supplementary Figure 2. Variant sites in carrier mothers of BMD patients. **(A)** Distribution of ends of deletions in carrier mothers of BMD patients. **(B)** Distribution of duplications in carrier mothers of BMD patients. Horizontal bars represent the duplicated regions. Gray and black bars represent carrier mothers with and without family history, respectively. **(C)** Distribution of small mutations. Gray and black symbols represent carrier mothers with and without family history, respectively. Squares, triangles, circles, and rhombus represent nonsense mutations, splice site mutations, small deletions/insertions, and missense mutations, respectively. The star represents a point mutation (c.-54T>A) in the 5′-untranslated region (UTR).
