# Supplementary figures and images for: Comparison of Carrier and de novo Pathogenic Variants in a Chinese DMD/BMD Cohort
Source: Front Neurol. 2021 Aug 5;12:714677. doi: 10.3389/fneur.2021.714677 (PMC8375267; doi:10.3389/fneur.2021.714677)

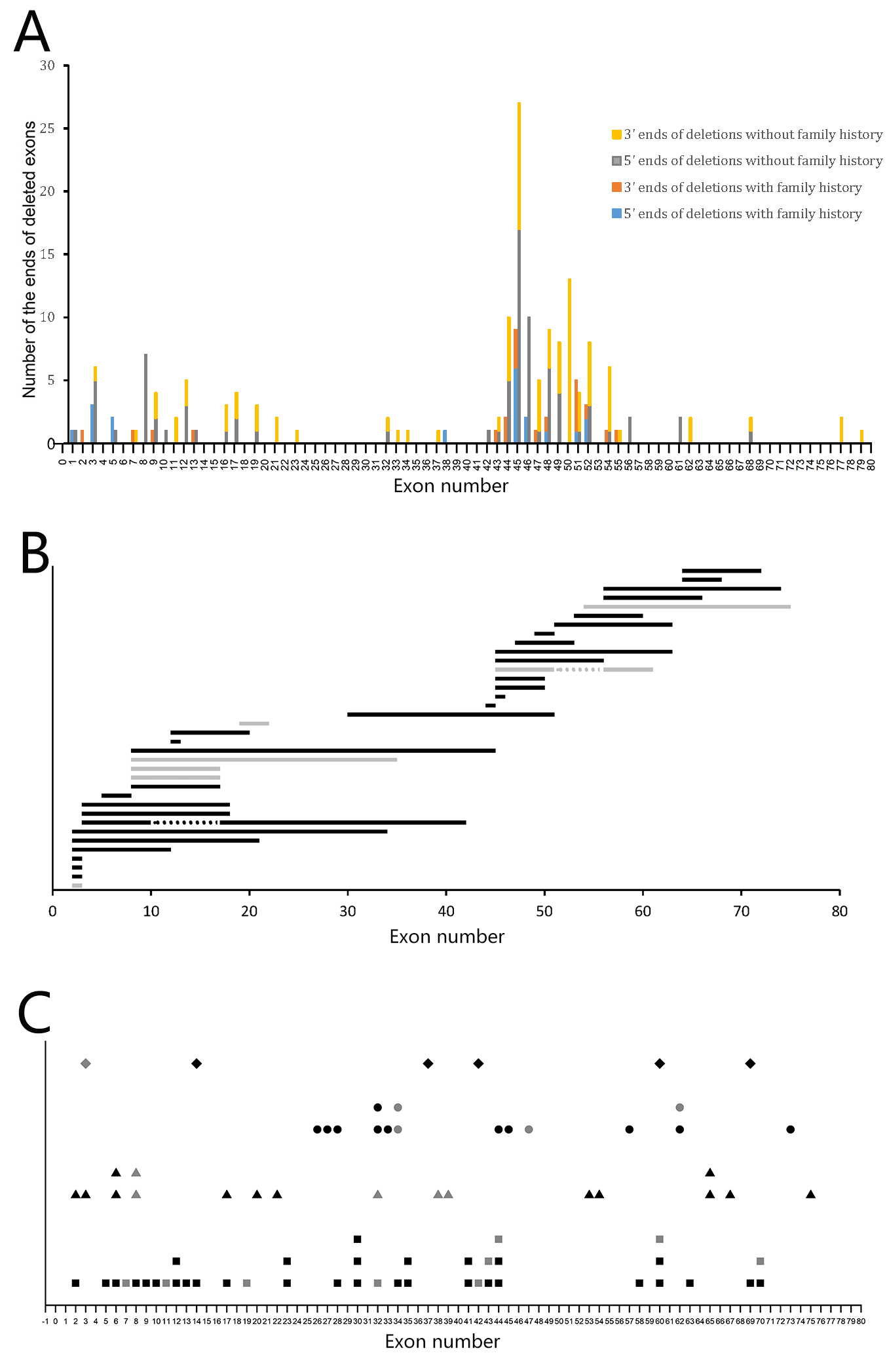

Supplement: Supplementary file 2 [file Image_1.TIF]

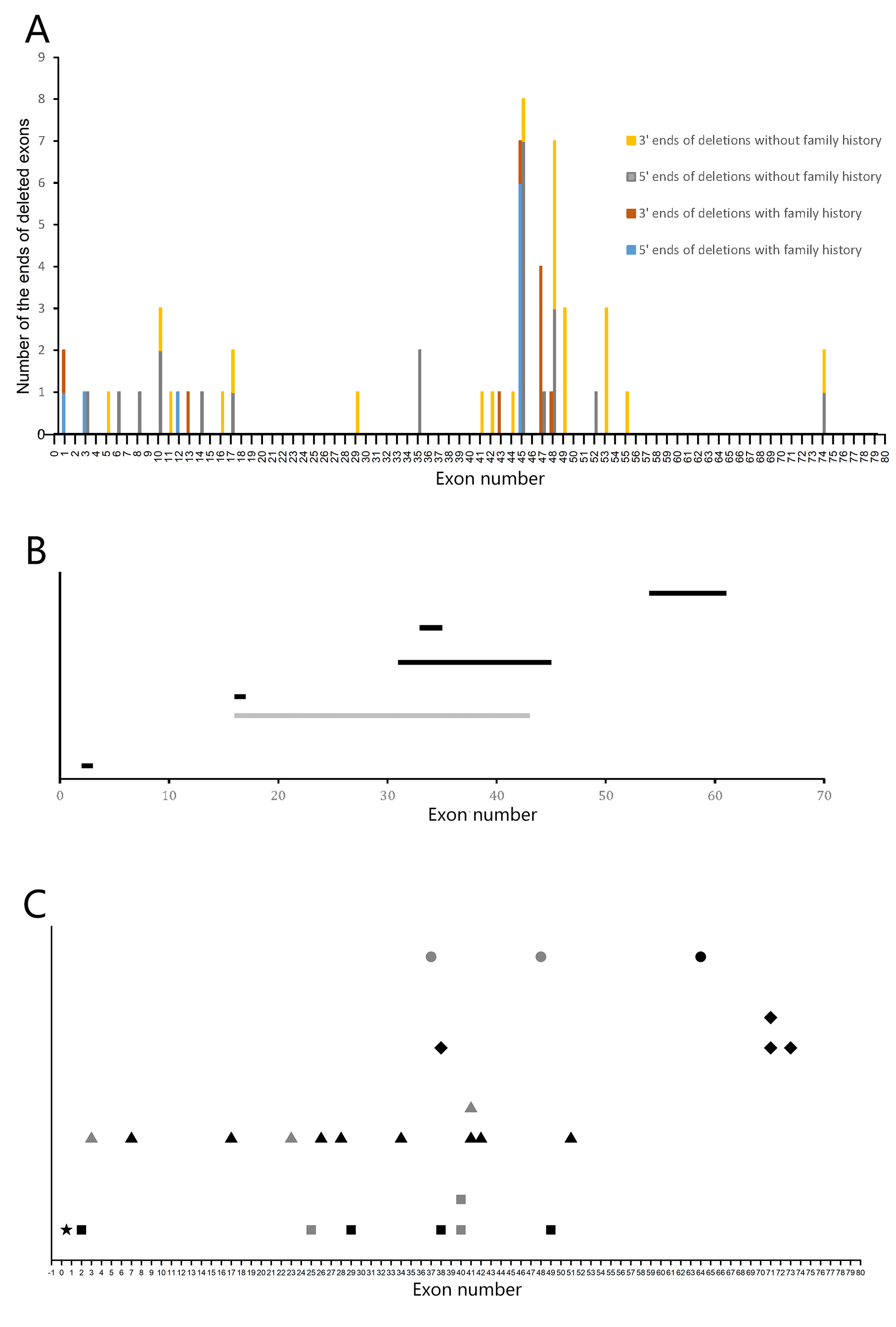

Supplement: Supplementary file 3 [file Image_2.TIF]
